# Inspiratory Muscle Rehabilitation Training in Pediatrics: What Is the Evidence?

**DOI:** 10.1155/2022/5680311

**Published:** 2022-08-18

**Authors:** Dharini M. Bhammar, Harrison N. Jones, Jason E. Lang

**Affiliations:** ^1^Center for Tobacco Research, Division of Medical Oncology, Department of Internal Medicine, The Ohio State University, Columbus, OH, USA; ^2^Department of Head and Neck Surgery & Communication Sciences, Duke University, Durham, NC, USA; ^3^Division of Pulmonary and Sleep Medicine, Duke Children's Hospital and Health Center, Durham, NC, USA

## Abstract

Pulmonary rehabilitation is typically used for reducing respiratory symptoms and improving fitness and quality of life for patients with chronic lung disease. However, it is rarely prescribed and may be underused in pediatric conditions. Pulmonary rehabilitation can include inspiratory muscle training that improves the strength and endurance of the respiratory muscles. The purpose of this narrative review is to summarize the current literature related to inspiratory muscle rehabilitation training (IMRT) in healthy and diseased pediatric populations. This review highlights the different methods of IMRT and their effects on respiratory musculature in children. Available literature demonstrates that IMRT can improve respiratory muscle strength and endurance, perceived dyspnea and exertion, maximum voluntary ventilation, and exercise performance in the pediatric population. These mechanistic changes help explain improvements in symptomology and clinical outcomes with IMRT and highlight our evolving understanding of the role of IMRT in pediatric patients. There remains considerable heterogeneity in the literature related to the type of training utilized, training protocols, duration of the training, use of control versus placebo, and reported outcome measures. There is a need to test and refine different IMRT protocols, conduct larger randomized controlled trials, and include patient-centered clinical outcomes to help improve the evidence base and support the use of IMRT in patient care.

## 1. Introduction

Pulmonary rehabilitation (PR) is a class of treatments that aims to help patients suffering from chronic respiratory symptoms [1]. PR usually employs a combination of modalities that improves fitness, disease understanding, and lifestyle choices in ways that promote overall health and improved quality of life [1]. PR is most commonly prescribed for chronic obstructive pulmonary disease (COPD); however, PR can be effective in a number of other conditions, particularly those associated with dyspnea and fatigue, such as heart disease, interstitial lung disease, lung cancer, cystic fibrosis, and asthma [2–6]. To date, PR is rarely prescribed in pediatric conditions and may very well be underutilized.

In most cases, PR is prescribed by a physician and leverages the expertise and guidance of physical and respiratory therapists [7]. PR has the advantage of being practical in numerous settings and can be beneficial with a range of frequencies, ranging from daily to just a few times per month. PR involves a combination of whole-body exercise (e.g., walking, aerobics, cycling, and swimming) and skeletal muscle resistance or strength training to build strength and endurance of the heart, limb muscles, and respiratory muscles. PR can also include various forms of breathing exercises, including those that improve the strength and endurance of the respiratory muscles. Altogether, PR can relieve respiratory symptoms, reduce dyspnea, and increase mobility, endurance, and disease-related quality of life [8–11].

## 2. Respiratory Musculature

### 2.1. Mechanics of Breathing

The lungs cannot fulfill their gas exchange role without a mechanical apparatus (or pump) that expands the lungs and draws air into the lower airways. This complex apparatus includes the thoracic cage (made up of the spinal vertebrae, the ribs, and sternum), the lung pleura, and the respiratory muscles. The respiratory muscles are similar in nature to other skeletal muscles, and during quiet breathing, they work in unison to expand the thoracic cage and induce inhalation. Respiratory muscles have both voluntary and involuntary innervation and are thought to have greater resilience to fatigue than other skeletal muscles. The muscular diaphragm is the main driver of inhalation. During quiet involuntary breathing, the diaphragm mostly acts alone. As the diaphragm contracts, it flattens and descends into the abdominal cavity, leading to drops in intrapleural and alveolar pressures. Assuming airway patency, air moves from the atmosphere to the lower, subatmospheric pressures in the alveoli.

Exhalation, on the other hand, is a more passive process and results from the relaxation of the respiratory muscles. The diaphragm relaxes and ascends, allowing recoil of the lungs and thoracic cage to the resting volume known as functional residual capacity (FRC). During hyperventilation or volitional exhalation, the rectus abdominal muscles (and to some degree the internal intercostals, intercostales intimi, and subcostal muscles) promote thoracic recoil and forced exhalation. During volitional breathing or hyperventilation induced by exercise, exhalation is facilitated mainly by the abdominal muscles (rectus and transversus abdominal muscles and internal and external oblique muscles) and secondarily by the internal intercostals. Contraction of these muscles together actively augment the ascent of the diaphragm, raising the pleural and alveolar pressures and leading to rapid exhalation.

### 2.2. Developmental and Pediatric Specific Issues with the Diaphragm and Respiratory Musculature

Key developmental issues are involved in inspiration during health and disease. All respiratory muscles originally derive from embryonic (cranial and trunk) mesoderm. The embryonic and early-life development of the diaphragm is critical to normal respiration and can exhibit developmental defects that impair breathing efficiency. The muscular diaphragm develops as a thin sheet that is both a central tendon and emanating muscle connecting anteriorly to the costal cartilages of ribs 7–10 and xiphoid and posteriorly to the lumbar vertebral bodies, transverse processes, and the 11^th^ and 12^th^ ribs; it separates the thoracic from the abdominal cavity. After the diaphragm is fully developed, the descent of the diaphragm during contraction stabilizes and elevates the lowest ribs via its anterior connections. This is known as the bucket handle effect. It is thought that children have less efficient inspiratory motion due to incomplete diaphragmatic development [12, 13], which can lead to earlier fatigue and resulting dyspnea with activity. In addition, the increased thoracic compliance seen in children further makes inspiration less efficient. With respiratory disease or when the body is under stress (i.e., during exercise), increased work of breathing and greater dyspnea can result. Additionally, the pediatric diaphragm sits more superiorly in the thoracoabdominal cavity with a reduced zone of apposition. Children's ribs are more horizontally aligned preventing a more efficient bucket-handle effect, raising the ribs up and out.

### 2.3. Respiratory Muscle Fatigue

Several factors can predispose respiratory muscles to fatigue in health and disease. Any factor that increases the energy demands of respiration impairs the capacity of the respiratory muscles to handle an increased ventilatory workload and quickly leads to respiratory muscle fatigue and dyspnea. Two common examples include restrictive lung disease (e.g., obesity, myopathy) and airway collapse and hyperinflation (e.g., asthma), both of which limit the respiratory muscles from handling increased ventilatory workload. Secondly, reduced energy reserves, malnutrition, or homeostatic problems, such as circulating acidosis or reactive oxygen species, can impair respiratory muscle function leading to fatigue. Lastly, deconditioning or atrophy (following severe illness or after prolonged mechanical ventilation), anemia, low cardiac output states, and hypoxemia all predispose to diaphragmatic dysfunction and fatigue.  Table 1 shows the most common conditions that can affect the functioning of the respiratory muscles in childhood.

### 2.4. Assessment of Respiratory Muscle Functioning

More research is needed to understand how best to measure and follow changes in respiratory muscle functioning. The strength of the respiratory muscles can be quantified in the clinical setting easily by the maximum inspiratory pressure (MIP) at the mouth by manometry against a closed mouthpiece [14]. These tests require patient cooperation and maximal effort. Current guidelines recommend at least three inspiratory maneuvers with maximal effort within 10%. Typically, the MIP is measured at the initiation of inspiration from the lung's residual volume. Maximal expiratory pressure (MEP) measured at expiration from total lung capacity measures the strength of the expiratory muscles [15]. Some conditions such as obesity do not demonstrate uniformly reduced baseline inspiratory muscle strength (i.e., MIP) but display greater fatigue of respiratory muscles under stress. Though resting MIP is not a direct measure of fatigue, it is closely correlated with inspiratory muscle fatigue and exertional dyspnea [15–17]. Currently, there are no universally accepted measures of inspiratory muscle endurance or fatigue. Despite this, several procedures have been described in the research setting, but all have considerable limitations. Procedures that have been described but need further pediatric validation include various forms of incremental-load testing, constant-load testing, and time trials [14, 15].

### 2.5. Pediatric Methods for Training the Inspiratory Muscles

Training approaches for inspiratory muscles can broadly be separated into strength-or endurance-training approaches. Strength-training approaches generally involve higher external loads (intensity) with lower training volumes, while endurance-training approaches typically use lower external loads and higher training volumes [18]. Although training methods vary considerably, three primary methods have been used to train the respiratory muscles: (1) voluntary isocapnic hyperpnea (VIH), (2) flow-resistive loading, and (3) pressure-threshold loading.

#### 2.5.1. VIH Training

VIH training typically requires vigorous hyperventilation for up to 30 minutes to target respiratory muscle endurance. To prevent hypocapnia, incorporation of a dead space rebreathing circuit or supplemental oxygen to avoid hypoxemia and maintain isocapnia is necessary. Training sessions generally occur at a frequency of 3–5 times per week at an intensity of 60–90% of maximal voluntary ventilation (MVV). A limitation of VIH is that high motivation is required as patients may find training to be strenuous and time-consuming [18–21]. Overall, VIH appears to enhance measures of respiratory muscle endurance but not strength [18, 22].

#### 2.5.2. Flow-Resistive Loading

**Figure 1:**
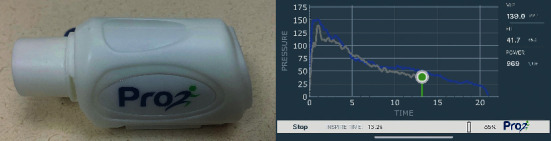
PrO_2_™ IMRT device (left) with user biofeedback (right) in PrO_2_™ Fit app via Bluetooth connection.

Flow-resistive loading provides the respiratory muscles an external load during ventilation through a variable diameter orifice. Traditionally, this is accomplished with the use of analog devices with adjustable dials, which allow the orifice surface area to be manipulated to change the flow and resulting load. For a given flow rate, the load increases as the orifice surface area decreases, although load also varies with flow rate. Therefore, in order to provide a quantifiable load, respiratory flow or pressure must be measured, which has led to the development of devices that provide electronic flow-resistive loading. For example, the K-series devices from POWERbreathe® provide an external load via an electronically controlled orifice that varies to achieve a target load or flow rate. Similarly, the PrO2™ device (Figure 1) provides a respiratory load via a fixed 2 mm port, incorporates an integrated pressure transducer to quantify resistance, and uses a Bluetooth-connected smartphone app to provide targeted loads, biofeedback, and user adherence data.

#### 2.5.3. Pressure-Threshold Loading

**Figure 2:**
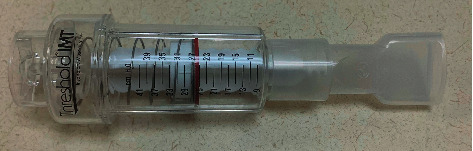
Threshold® IMT from Phillips Respironics provides pressure-threshold loading from 9 to 41 cm H_2_O resistance.

**Figure 3:**
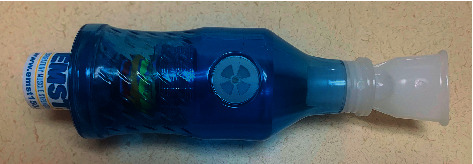
EMST150^TM^ with IA150^TM^ from Aspire Products provides pressure-threshold loading from 30 to 150 cm H_2_O resistance.

Pressure-threshold loading provides an external load to the respiratory muscles via ventilation through an orifice that remains closed with a spring-loaded valve or other methods until a target pressure is achieved (e.g., Threshold® IMT from Phillips Respironics (Figure 2), EMST150™ with IA150™ from Aspire Products (Figure 3)). This approach allows delivery of a quantifiable and adjustable external load that is relatively independent of flow rate using simple and relatively inexpensive equipment. A limitation is that once the target pressure is achieved, the training resistance remits. Additional details about training devices including commercial product examples, strengths, and limitations can be found in Table 2.

The purpose of this narrative review is to summarize the currently available literature on the various types of IMRT protocols utilized in pediatric populations including youth athletes, children with neuromuscular disease, children with asthma, and children with obesity and to summarize the effectiveness of IMRT protocols on pulmonary and performance outcomes.

## 3. Effectiveness of Inspiratory Muscle Rehabilitation Training

### 3.1. Description of Literature Search

A PubMed search was performed using the search terms “inspiratory training” or “inspiratory muscle training” AND “children” or “child” or “pediatric” AND the appropriate population of interest such as “athletes,” “neuromuscular disease,” “obesity,” or “asthma” between January and March 2022. By screening the title and abstract, we were able to identify original prospective studies with full text available in English in children <18 years that included an IMRT intervention that was described in sufficient detail so as to be reproducible. IMRT was defined as an intervention that involved loading the inspiratory muscles with an external device to improve inspiratory muscle strength and/or endurance. The references and citations of each article identified with the search were also screened. Study quality was assessed using the National Heart, Lung, and Blood Institute's tailored quality assessment tools [23] with individual study quality assessments provided in Supplemental Tables 1–3.

### 3.2. IMRT in Pediatric Athletes

We performed a review of the literature on the use of inspiratory muscle training in pediatric-age athletes (Table 3). Most pediatric IMRT studies have involved swimmers [24–28], including one in disabled swimmers [29]. One study was on soccer players [30]. Wells and colleagues examined the effects of six weeks of moderate-intensity IMRT followed by six weeks of high-intensity IMRT compared with six weeks of sham (or placebo) followed by six weeks of moderate-intensity IMRT in national-level competitive swimmers [24]. They reported improvements in MIP and MEP as well as inspiratory and expiratory muscle power output during the respiratory muscle endurance test after 12 weeks in both groups with no differences between the groups. In this study, there was no effect of IMRT on dyspnea or swim performance assessed with an incremental swimming test protocol. The increase in MIP after IMRT was consistent among the studies in swimmers [25–29] and in the study in soccer players [30].

Although the performance was not improved after IMRT in the national-level competitive swimmers, IMRT improved 50-meter and 200-meter swim performance in short- and middle-distance swimmers training 20 hours/week [26], 100-meter and 200-meter performance in swimmers training 10 hours/week but not in those training 14–19 hours/week [27], and apnea max (maximum distance swum after inspiration) in fin-swimmers [28]. Running performance was improved in soccer players after IMRT, more so than in the control training group [30]. Ratings of perceived exertion and dyspnea were reportedly lower after IMRT in swimmers [25, 26]. Forced vital capacity (FVC) and forced expiratory volume in one second (FEV_1_) improvements were noted in most [24, 26, 29, 30] but not all [25, 28] studies that included spirometry.

### 3.3. IMRT in Children with Neuromuscular Diseases

We performed a review of the literature on IMRT in children with neuromuscular diseases. Several studies were excluded due to insufficient details regarding the specifics of their training protocol or outcomes [31–38]. Of the eight included studies (Table 4), five included children with muscular dystrophy, of which four involved children with Duchenne muscular dystrophy (DMD) [39–42] and one involved children with limb girdle muscular dystrophy (LGMD) or spinal muscular atrophy (SMA) [43]. In the four studies with DMD patients, respiratory training occurred frequently from twice daily to 5 times per week for an overall duration of 5–6 weeks. Topin and colleagues [41] and Takaso and colleagues [42] both used pressure-threshold loading at an intensity of 30% MIP. DiMarco and colleagues [39] and Martin and colleagues [40] both used flow-resistive loading developed in-house at an intensity that could be tolerated for 5–15 minutes in the former or ventilation to exhaustion in less than 3 minutes in the latter. Training intensity was defined temporally by DiMarco et al. (15–20 minutes), while Martin and colleagues required 3 efforts to exhaustion. Additionally, Martin et al. required subjects to complete maximum isometric inspiratory/expiratory maneuvers over their range of vital capacity for 30 minutes. Overall, based on their training intensity and volume, these 4 studies in DMD seemed to target respiratory muscle endurance more than strength, except for the study from Martin and colleagues that specifically targeted both strength and endurance.

Improvements in inspiratory muscle endurance were noted in all three of the studies which included endurance measures including improved MVV [39], the duration that MIP/MEP maneuvers could be sustained ≥90% [40], and time-to-exhaustion testing [41]. Inspiratory muscle strength (MIP) did not improve in three of the studies [39–41]. Takaso et al. [42] reported improvements in forced vital capacity. Yeldan et al. [43] studied inspiratory pressure-threshold loading 2 times per day for 12 weeks in patients with LGMD and SMA. Training intensity was 30% MIP, and volume was 15 minutes/session. Based on MIP, inspiratory muscle strength improved, though FVC and FEV_1_ were unchanged.

The population of interest in the three remaining studies were children with infantile-onset Pompe disease (IOPD) including a before-and-after trial in 9 subjects [44] and 2 case studies involving three participants [45, 46]. Smith et al. [44] used inspiratory pressure-threshold loading twice per week for 90 days. The intensity was the highest tolerated load in which they generated at least 50% of their unassisted tidal volume for a volume of 3–4 sets of 6–10 repetitions. Jones et al. [45] and Crisp et al. [46] used inspiratory and expiratory pressure-threshold loading 5 times per week for 12 weeks. Training intensity was 60–70% MIP/MEP and volume was 3 sets of 25 inspiratory and expiratory repetitions. Results from Smith et al. [44] differed based on the mechanical ventilation status of participants. For those on full mechanical ventilation, no changes in peak inspiratory flow, tidal volume, inspiratory time, and expiratory time were noted. For subjects on partial or no mechanical ventilation, peak inspiratory flow and tidal volume improved. In the case studies from Jones et al. [45] and Crisp et al. [46], increases in MIP, MEP, and peak cough flow were noted.

Overall, respiratory training appeared to improve respiratory muscle endurance and is likely to offer benefits in children with neuromuscular diseases considering the effects of respiratory muscle fatigue under stress on the morbidity and mortality in this population. Additionally, training appeared to be well-tolerated with no reported adverse events [39,43,45,46]. However, the lack of focus on patient-centered clinical end points in these studies highlights the need for additional research on these populations.

### 3.4. IMRT in Children with Airways Disease

A Cochrane review involving 115 adult patients was published in 2013 summarizing the results of 5 studies employing IMRT in patients with asthma [47]. The IMRT sessions in these 5 Cochrane studies lasted between 10 and 30 minutes, and IMRT regimen duration ranged from 3 to 12 weeks. IMRT was consistently well-tolerated and consistently improved inspiratory strength measured by MIP. However, definitive clinical conclusions regarding asthma outcomes were not possible due to the relatively small number of subjects and heterogeneous end points across studies. This Cochrane review included two studies in which IMRT increased inspiratory muscle strength in adults with mild [48] and mild-to-moderate asthma [49] who were high consumers of inhaled *β*_2_ agonists (>1 puff/day) for breakthrough asthma symptoms. The increased inspiratory muscle strength has seen following IMRT correlated closely with a decrease in dyspnea with exercise (measured by Borg scoring) and a decrease in *β*2-agonist consumption [49]. Since this 2013 review, two additional IMRT studies in adults with asthma (*n* = 81) demonstrated reduced dyspnea, respiratory symptoms, and activity impairment with IMRT [50, 51]. These data emphasize the promise that inspiratory muscle rehabilitation has for patients with asthma with excess dyspnea and high *β*_2_-agonist bronchodilator use. There were no studies describing exacerbation events that required the use of reliever medication, emergency department visits, or hospital admissions. This 2013 Cochrane review strongly recommended that studies on children be conducted in the future.

To date, only two IMRT studies have been published in pediatric asthma [52, 53], which were not included in the Cochrane review (Table 5). The first, by Lima and colleagues, was a randomized controlled study involving 50 children with asthma allocated to 1 of 2 groups: an IMRT group, comprising 25 children, and a control group, comprising 25 children who submitted to monthly medical visits and education on asthma [52]. The IMRT was performed using a pressure threshold load of 40% of MIP. All children completing IMRT spent the first 25 minutes doing diaphragmatic breathing, fractionated breathing, and pursed-lip breathing sitting and in the supine position (each exercise was performed in a series of 10 repetitions). Next, pressure-threshold loading IMRT was performed for 25 minutes using a spring-loaded adjustable resistance valve device. During the first 20 minutes, inspirations were performed repeatedly in a series of 10 repetitions for 60 seconds each, separated by 60 second rest periods. During the final 5 minutes, IMRT was performed continuously without rest. The IMRT resistance load was 40% of MIP, which was calculated at the beginning of each session. The active intervention lasted for 7 weeks. The 25 participants in each intervention group were generally similar. The mean age in both groups was just under 10 years. At 49 and 90 days, the IMRT group had significantly improved MIP, MEP, and PEF from baseline, which were significantly improved over that of the control group. Children undergoing IMRT experienced a mean 127% improvement from baseline in MIP (standardized effect size (SES) improvement of 10.7) at 90 days and 76% improvement from baseline in PEF (SES improvement of 2.6) at 90 days. Once IMRT was discontinued, improvements in MIP, MEP, and PEF remained stable after 7 weeks. There were clear numeric reductions from baseline in the frequency of asthma attacks, nocturnal and diurnal symptoms, and reported activity limitation in IMRT-treated children not seen in the control arm.

More recently in 2020, Elnaggar investigated the efficacy of IMRT on lung function, respiratory muscle strength, and asthma symptoms in asthmatic children [53]. Elnaggar used a randomized placebo-controlled assessor-blinded study of 34 children with asthma and randomized participants to receive either the IMRT at 40% of the MIP for 20 minutes/session, 3 times per week, over 12 consecutive weeks (IMRT group; *n* = 17) or placebo IMRT at 5% of MIP (placebo group; *n* = 17). Both interventional groups received a conventional respiratory rehabilitation program. The researchers reported significant post-treatment differences between the IMRT and placebo groups in FEV_1_ (*P* = 0.003), FVC (*P* = 0.001), FEV_1_/FVC (*P* = 0.004), MIP (*P* = 0.002), and MEP (*P* = 0.004), adjusted to the pretreatment values, in favor of the IMRT group. In addition, IMRT was associated with significant improvement in asthma symptom scores measured by the validated Asthma Control Test (ACT) (*P* = 0.001). Elnaggar concluded that the incorporation of IMRT in children with asthma can improve respiratory function, enhance respiratory muscle strength, and improve children's perception of asthma symptoms.

### 3.5. IMRT in Children with Obesity

Studies that have examined the effects of IMRT in overweight and obese adults have reported improved exercise performance [54–57], MIP [56–58], respiratory muscle endurance [54, 55], and reduced exertional dyspnea [54, 55]. Three studies have examined the effects of IMRT in adolescents with obesity, all from the same research group in Italy [59–61] (Table 6). The IMRT protocol was completed at 50–60% MVV, 1 session per day, 5 days per week, with each session lasting 12–18 minutes. LoMauro et al. reported significant weight loss (3.5 kg), improvements in exercise performance and FVC, and reduced dyspnea and leg discomfort during exercise after a 3-week multidisciplinary body weight reduction program that included IMRT [61]. However, since the program included diet, aerobic exercise, and psychological and nutrition counseling in addition to the IMRT component, it is impossible to tease out the independent effects of IMRT. In two studies that differed on the mode of exercise (i.e., cycling vs. walking), three weeks of IMRT combined with a multidisciplinary weight loss program was effective in reducing the oxygen cost of exercise during moderate-intensity walking and vigorous-intensity walking and cycling when compared with a multidisciplinary weight loss program alone; both interventions resulted in similar weight loss [59, 60]. IMRT combined with a multidisciplinary weight loss program also resulted in improved peak performance, reduced exertional dyspnea ratings, higher FVC, and a reduced slow component of oxygen uptake during vigorous-intensity exercise [59, 60]. The slow component of oxygen uptake during vigorous-intensity exercise is partly due to the increased oxygen cost of breathing [62], and a greater slow component can be associated with reduced exercise tolerance [63]; improvements in the slow component of oxygen uptake could therefore have beneficial implications for exercise tolerance for the pediatric obese population.

## 4. Clinical Considerations

IMRT appears to represent a viable non-pharmacologic option for patients in need of improving their exercise tolerance and reducing dyspnea. The exact mechanisms by which IMRT reduces asthma symptoms remain poorly defined. For children with asthma, we speculate that IMRT improves disease control through improved lung function, reduced airway closure, and reduced respiratory muscle fatigue leading to reduced frequency of dyspnea, which previously was perceived as asthma symptoms. While there have not been any published reports on the effectiveness of IMRT in children with obesity and asthma, a special population that experiences a high symptom burden [64], favorable results showing that IMRT improves performance and reduces dyspnea in nonasthmatic children with obesity are encouraging. IMRT has major advantages: it is simple, safe, and inexpensive, and is generally very attractive to many patients interested in a nonpharmacologic therapy. For patients who experience significant dyspnea or exercise intolerance with consequent physical activity avoidance, IMRT could be a precursor to the implementation of a home- or center-based exercise program. A careful assessment by a healthcare professional to evaluate differential diagnoses for dyspnea and/or exercise intolerance, a baseline test of inspiratory muscle strength, and a cardiopulmonary exercise test could help identify patients who have reduced inspiratory muscle strength and/or impaired breathing mechanics; these are the patients that may benefit most from IMRT. Follow-up testing, regular contact between the healthcare professional and patient, and documentation of the effectiveness of IMRT through clinical audits could inform clinical practice models [65].

## 5. Future Directions

Relatively few children have been formally studied with high-quality IMRT utilizing randomization and blinding. Among studies included in this review, approximately half included both inspiratory and expiratory muscle training. It may be reasonable to hypothesize that expiratory muscle training would improve expiratory muscle strength (i.e., MEP). Although this hypothesis was supported by two randomized trials [26, 28] and two case reports [45, 46], it was not supported by two other randomized trials [24, 40]. In addition, three studies on obese youth included an inspiratory and expiratory training component but did not measure inspiratory or expiratory muscle strength [59–61]. Interestingly, three randomized trials with an exclusive inspiratory training protocol nevertheless reported improvements in MEP [53]. Whether there is any additional benefit to including an expiratory muscle training component in pulmonary rehabilitation protocols remains unclear. Theoretically, since the inspiratory phase of breathing is active while the expiratory phase is mostly due to passive recoil except at higher ranges of ventilation, the benefit of training expiratory muscles is not readily apparent. However, studies that compare inspiratory training alone versus combined training in different youth populations may offer insights into this issue and inform interventions in the PR field. The optimal dose of IMRT regarding intensity (percent of MIP), repetitions and weekly frequency, and duration are poorly understood. Dose ranging and physiologic studies that describe potential mechanisms are needed to guide clinical practice, as are studies that include patient-centered clinical end points. Larger randomized controlled trials that evaluate IMRT effectiveness and look at mechanistic outcomes are also needed in the pediatric population in addition to trials that can offer insight into specific patient groups that may benefit most from IMRT.

## 6. Conclusion

Dyspnea and exercise intolerance are common symptoms noted in pediatric patients with neuromuscular diseases, asthma, and obesity. Only a handful of studies have investigated the effects of IMRT on clinical outcomes and underlying physiological mechanisms in these pediatric patients. These studies demonstrate that IMRT is likely effective in improving respiratory muscle strength and reducing airway closure (i.e., higher FVC) and potentially in improving breathing mechanics and reducing dyspnea during exercise. These mechanistic changes can help explain improvements in symptomology and clinical outcomes, and they highlight our evolving understanding of the role of IMRT in pediatric patients.

## Figures and Tables

**Table 1:** Causes of respiratory muscle weakness or fatigue.

Common classes	Examples/comments
Injury to spinal cord/diaphragmatic innervation	Trauma affecting spinal nerves C3–5
Arnold–Chiari malformations	Types I–IV
Guillain–Barre syndrome	Following acute infections, rarely vaccines
Myasthenic syndromes	
Muscular dystrophies	Duchenne, Becker, Myotonic, Limb-girdle
Spinal muscular atrophy	Types 1 (infantile onset), 2 (intermediate), 3/4 (mild, adult onset)
Multiple sclerosis	
Metabolic myopathy	Pompe, McArdle, mitochondrial
Airway diseases	COPD, asthma
Diaphragmatic disorders	Congenital diaphragmatic hernia, eventration
Cerebral vascular accidents (stroke)	
Critical illness	Prolonged ECMO, chronic mechanical ventilation
Inflammatory myopathy	Polymyositis, dermatomyositis
Iatrogenic (steroid myopathy, radiation injury)	
Poisoning/toxins	Botulism, narcotics
Infections	Polio, polio-like viruses, acute flaccid myelitis
Obesity	Restricted diaphragmatic motion and thoracic expansion

*Note.* COPD – chronic obstructive pulmonary disease and ECMO – extracorporeal membrane oxygenation.

**Table 2:** Types of inspiratory muscle rehabilitation training.

Device type	Subtypes	Commercial products	Strengths	Limitations
Voluntary isocapnic hyperpnea	N/A	SpiroTiger	Real-time biofeedback	Relatively expensive and complex equipment Limited portability Training is strenuous and time-consuming

Flow-resistive loading	Analog	Breather, P-Flex	Inexpensive Highly portable	Load varies with flow rate Limited biofeedback capability
	Digital	POWERbreathe K-series	Highly programmable Automated data collection Real-time biofeedback with Breathe-Link software for K4/K5 KH1 allows measurement of MIP Wide range of resistance (5–200 cm H_2_O)	Relatively expensive Full functionality for K4/K5 devices requires a computer, which increases cost and decreases portability
		PrO2	Highly portable Highly programmable Automated data collection Real-time biofeedback provided via smartphone app Measurement of MIP	Relatively expensive Full functionality requires a smartphone/tablet and an app

Pressure-threshold loading	N/A	Threshold IMT	Inexpensive Highly portable Load is relatively independent of flow rate Calibration markings are easy to interpret and set	Limited biofeedback capability Available load is low range and narrow (9–41 cm H_2_O)
		Analog devices from POWERbreathe (e.g., Medic, Classic, Plus)	Inexpensive Highly portable Load is relatively independent of flow rate Relatively wide range of loads available	Limited biofeedback capability Can be difficult to determine range of load for various models Ordinal calibration markings do not quantify load
		EMST75/150 with IA150	Relatively inexpensive Highly portable Load is relatively independent of flow rate (mm) Relatively wide range of loads available (5–75 cm H_2_O with EMST75; 30–150 cm H_2_O with EMST150)	Limited biofeedback capability For inspiratory training, both expiratory device (EMST75 or EMST150) and inspiratory adapter (IA150) are required Calibration markings to quantify load may be difficult to interpret

*Note.* MIP – maximal inspiratory pressure and EMST – expiratory muscle strength training.

**Table 3:** Inspiratory muscle rehabilitation training (IMRT) in youth athletes.

Citation	Study Design	Population	Intervention	Outcomes	Limitations
Wells et al. 2005 [24]	RCT	National-level competitive swimmers, 15.6 ± 1.3 years *n* = 34 (20 females, 14 males)	Duration: 12 weeks	At 6 weeks, no change in MIP and MEP in IMRT or sham groups; at 12 weeks, ∆MIP = 14.4 cmH_2_0 and ∆MEP = 20 cmH_2_0 in females (combining IMRT and sham subjects) with no changes in males	No statistical between-group comparisons reported
			Frequency: 10 sessions/week	MVV_15_, FEV_1_, and FVC increased in both IMRT and sham groups after 12 weeks; no difference between IMRT and sham at 6 weeks	
			Intensity: 50% weeks 1–3 and 60% weeks 3–6 for MIP and MEP; 70% weeks 7–9 and 80% weeks 10–12 for MIP and MEP	No differences in performance (swim velocity) or dyspnea	
			Volume: 10 breaths per session		
			Type: inspiratory and expiratory flow-resistive loading		
			Equipment: PowerLung		
			Control: sham IMRT (10% MIP and MEP) for 6 weeks followed by moderate IMRT (50% weeks 7–9 and 60% weeks 10–12 for MIP and MEP)		
			(Both groups continued regular swim training; only first 6 weeks represent differences between IMRT and sham)		

Kilding et al. 2010 [25]	RCT	Club-level competitive swimmers *n* = 16 (6 females, 10 males)	Duration: 6 weeks	∆MIP = 10.5 cmH_2_0 in IMRT group vs. 0.3 cmH_2_0 in sham group	
			Frequency: 7 days/week	Swim time improved for 100 m and 200 m time trials, but not for 400 m time trial	
			Intensity: 50% MIP. Instructed to increased load periodically so that 30 breaths could only just be completed	Rating of perceived exertion decreased across a range of intensities	
			Volume: 30 breaths, twice a day	FVC, FEV_1_, PEF: No change	
			Type: pressure threshold loading	MEP not reported	
			Equipment: POWER-breathe		
			Control: sham IMRT, 60 slow protracted breaths once daily at 15% MIP		

Lemaitre et al. 2013 [26]	Controlled before and after study	Swimmers, 13–18 yr, avg. training 45–48 wk/yr, 20 h/wk *n* = 20 (7 females, 13 males)	Duration: 8 weeks	∆MIP = ≈25 cmH_2_0 in RMET group vs. no change in control group	MIP and MEP reported in figures (absolute values not reported)
			Frequency: 5 days per week	∆MEP = ≈25 cmH_2_0 in RMET group vs. no change in control group	
			Intensity: 60% of MVV_12_	Competition swim time on 50 m and 200 m improved	
			Volume: 30 min	Respiratory endurance test breathing duration increased from 16 to 24.6 min	
			Type: voluntary isocapnic hyperpnea (respiratory muscle endurance training or RMET)	*V*_Emax_ and maximum breathing frequency increased	
			Equipment: SpiroTiger	Rating of perceived exertion and rating of perceived dyspnea reduced	
			Control: usual training only	during the 50 m and 200 m race	
				FVC (% pred) and MVV increased	

Lomax et al. 2019 [27]	RCT	Swimmers, two groups based on training distance (low and high) *n* = 33 (15 females, 18 males)	Duration: 6 weeks	∆MIP = ≈55 cmH_2_0 (high-training IMRT group), ≈35 cmH_2_0 (low-training IMRT group) and ≈20 cmH_2_0 (high-training control group)	MIP reported in figures (pre and post absolute values not reported). 36% improvement in MIP (combined for high and low-training IMRT groups) reported in text of article
			Frequency: 7 days/wk	No change in MEP	
			Intensity: 50% MIP. Instructed to increased load periodically so that 30 breaths could only just be completed	100 m and 200 m swimming times improved in the low-training IMRT group only	
			Volume: 30 breaths, twice a day		
			Type: pressure threshold loading		
			Equipment: POWER-breathe		
			Control: usual swim training only		

Okrzymowska et al. 2019 [29]	RCT	Disabled swimming division athletes, 16–20 yr *n* = 16 (8 females, 8 males)	Duration: 8 weeks	∆MIP = 33 cmH_2_0 (IMRT group) and 16 cmH_2_0 (control group)	
			Frequency: 5 days/week	FVC, FEV_1_, and PEF increased in IMRT group	
			Intensity: 30% MIP in week 1, increased to 40% in weeks 2 and 3, 50% in weeks 4 and 5, and 60% in weeks 6–8	MEP increased in both groups	
			Volume: 30 breaths, 5 min in week 1 increased to 15 min in week 8, twice a day		
			Type: pressure threshold loading		
			Equipment: philips respironics		
			Control: usual swim training only		

Vašíčková et al. 2017 [28]	Randomized controlled trial (parallel arm with control group receiving the IMRT intervention after a 1-month washout)	Club-level fin-swimmers *n* = 20 (did not report sample size by sex)	Duration: 4 weeks	MIP increased by 20.8% in the IMRT group vs. 1.5% in the control group (post 4 weeks)	Results reported as median values; absolute values not reported for MIP and MEP
			Frequency: 7 days/week	MEP increased by 10.6% in the IMRT group vs. 5.1% decrease in the control group (post 4 weeks)	
			Intensity: 30% MIP and MEP and increased by 2 cm H20 every week until maximum possible resistance on the threshold devices	Length able to swim for one inspiration increased by 27.4% (IMRT in first phase), 20.7% (IMRT in second phase)	
			Volume: 10 maximal inspirations and 10 maximal expirations (strength) + 15 min of continuous breathing against resistance (endurance)	FVC, FEV_1_, PEF: no change	
			Type: pressure threshold loading (inspiratory and expiratory)		
			Equipment: philips respironics		
			Control: usual swim training (with IMRT completed in second phase after washout)		

Mackala et al. 2019 [30]	RCT	Club-level competitive junior soccer players *n* = 16 males	Duration: 8 weeks	∆MIP = 44 cmH_2_0 (IMRT group) vs. 11 cmH_2_0 (control group)	
			Frequency: 5 days/week	∆MEP = 41 cmH_2_0 (IMRT group) vs. 4 cmH_2_0 (control group)	
			Intensity: 40% MIP in week 1, increased by 5% every week to 80% in week 8	Running test distance increased by 5% in IMRT group vs. 2.1% in the control group (both changes were statistically significant)	
			Volume: 5 repetitions in week 1 to 15 repetitions in week 8, each repetition was 45 s of IMRT followed by a 15 s break, twice a day	FVC improved in IMRT group	
			Type: pressure threshold loading	FEV_1_ improved in both groups	
			Equipment: philips respironics		
			Control: usual training only		

*Note*. RCT–randomized control trial, MIP–maximal inspiratory pressure, MEP–maximal expiratory pressure, MVV_12_–Maximal voluntary ventilation measured by assessing ventilation during maximal voluntary effort for 12 seconds and extrapolating to calculate maximal ventilation in liters per minute, FEV_1_–forced expiratory volume in 1 second, FVC–forced vital capacity, PEF–peak expiratory flow, and V_Emax_–maximal ventilation.

**Table 4:** Inspiratory muscle rehabilitation training (IMRT) in children with neuromuscular disease.

Citation	Study design	Population	Intervention	Outcomes
DiMarco et al. 1985 [39]	Before-and after-trial	Various NMD: DMD (*n* = 5) LGMD (*n* = 5) FSHMD (*n* = 1)	Duration: 6 weeks	MIP: no ∆
		*N* = 11 (did not report sample size by sex)	Frequency: 2 x/day	MEP: no ∆
			Intensity: Inspiratory resistance tolerated for >5 minutes and <15 minutes	VC: no ∆
			Volume: 15–20 minutes/session	Maximum voluntary ventilation (MVV):
			Type: inspiratory flow-resistive loading	30% MVV: ↑128 ± 81%, p<0.01
			Equipment: developed in-house	50% MVV: ↑107 ± 36%, *p* < 0.01
				70% MVV: ↑85 ± 40%, *p* < 0.01
				90% MVV: ↑75 ± 24%, *p* < 0.01

Martin et al. 1986 [40]	Cross-over trial	DMD	Duration: 5 weeks	MIP: no ∆
		*N* = 18 males	Frequency: 5 days/week	MEP: no ∆
			Intensity: maximum isometric inspiratory/expiratory maneuvers at 20% intervals over VC range (strength training) or resistive IMT/EMT via various diameters of tubing that led to exhaustion in <3 minutes (endurance training)	VC: no ∆
			Volume: 30 minutes (strength training); ventilation to exhaustion 3 x (endurance training)	Inspiratory endurance (duration of MIP ≥90%): ↑ 8.5 ± 8.1 seconds, *p* < 0.01
			Type: isometric inspiratory/expiratory maneuvers against occluded tube (strength training);	Expiratory endurance (duration of MEP ≥90%): ↑ 6.1 ± 5.1 seconds, *p* < 0.01
			Inspiratory/expiratory flow-resistive loading (endurance training)	
			Equipment: developed in-house	

Topin et al. 2002 [41]	RCT	DMD	Duration: 6 weeks	MIP: no ∆
		*N* = 16 males	Frequency: 2 x/day	FVC: no ∆
			Intensity: 30% MIP	Inspiratory muscle endurance (*T*_lim_): ↑ from 307.6 ± 126.6 to 448.4 ± 176.7, *p* < 0.05
			Volume: 10 minutes/session	
			Type: inspiratory pressure-threshold resistance	
			Equipment: threshold IMT	
			(Control: sham-IMT 5% MIP)	

Yeldan et al. 2008 [43]	Non-RCT with alternating allocation	LGMD (*n* = 17), BMD (*n* = 6) recruited	Duration: 12 weeks	VC: no ∆
		*N* = 21 completed (8 females, 13 males)	Frequency: 2 x/day	FVC: no ∆
			Intensity: 30% MIP	FEV_1_: no ∆
			Volume: 15 minutes/session	MIP: ↑ 37.5 ± 22.8 cmH_2_0 (IMRT group) vs. 10.3 ± 12.1 cmH_2_0 (control group), *p* = 0.05
			Type: inspiratory pressure-threshold resistance	MEP: no ∆
			(Control: breathing exercises)	

Takaso et al. 2010 [42]	Before-and after-trial	DMD	Duration: 6 weeks	%FVC: increased from 21.5 ± 3.1 to 26.4 ± 2.8%
		*N* = 17 males	Frequency: daily	MIP and MEP not measured
			Intensity: 30% MIP	
			Volume: 3 sets of 15 repetitions	
			Type: inspiratory pressure-threshold resistance	
			Equipment: threshold IMT	

Jones et al. 2014 [45]	Case report	IOPD	Duration: 12 weeks	MIP: ↑ from 17 cmH_2_0 to 26 cmH_2_0 (in subject 1) and from 22 cmH_2_0 to 34 cmH_2_0 (in subject 2)
		*N* = 2 (1 female, 1 male)	Frequency: 5 x/week	MEP: ↑ from 16 cmH_2_0 to 20 cmH_2_0 (in subject 1) and from 43 cmH_2_0 to 80 cmH_2_0 (in subject 2)
			Intensity: 60–70% MIP/MEP	Peak cough flow (subject 2 only): ↑
			Volume: 3 sets of 25 inspiratory and expiratory repetitions	FVC: no ∆
			Type: inspiratory/expiratory pressure-threshold resistance equipment: threshold IMT, threshold PEP, EMST 150	

Smith et al. 2017 [44]	Before-and-after trial	IOPD 2–15-years-old	Duration: 12 weeks	Subjects on full MV: PIF, tidal volume, inspiratory time, expiratory time, duty cycle: no ∆
		*N* = 9 (did not report sample size by sex)	Frequency: 3 x/week	
			Intensity: highest tolerated load in which they generated at least 50% of unassisted tidal volume	Subjects on partial/no MV:
			Volume: 3-4 sets of 6–10 repetitions	↑ PIF at 5 cmH_2_O
			Type: inspiratory pressure-threshold resistance	Pre-post IMRT MIP not reported
			Equipment: threshold PEP, AccuPEEP	

Crisp et al. 2020 [46]	Case report	IOPD	Duration: 12 weeks	MIP: ↑ 10.1 cmH_2_O (after 12 weeks; 1^st^ treatment phase)
		*N* = 1 female	Frequency: 5 x/week	MEP: ↑ 35.2 cmH_2_O (after 12 weeks; 1^st^ treatment phase)
			Intensity: 60–70% MIP/MEP	Peak cough flow: ↑
			Volume: 3 sets of 25 inspiratory and expiratory repetitions	
			Type: inspiratory/expiratory pressure-threshold resistance	
			Equipment: threshold IMT, Threshold PEP, EMST 150	

*Note*. RCT–randomized controlled trial, NMD–neuromuscular disease, DMD–duchenne muscular dystropy, LGMD–limb-girdle muscular dystrophy, FSHMD–fascioscapulohumeral muscular dystrophy, MIP–maximal inspiratory pressure, MEP–maximal expiratory pressure, VC–vital capacity, IMT–inspiratory muscle training, EMT–expiratory muscle training, FVC–forced vital capacity, FEV_1_–forced expiratory volume in 1 s, BMD–Becker muscular dystrophy, IOPD–infantile-onset Pompe disease, MV–mechanical ventilation, PIF–peak inspiratory flow, and EMST–expiratory muscle strength training.

**Table 5:** Inspiratory muscle rehabilitation training (IMRT) in children with asthma.

Citation	Study design	Population	Intervention	Outcomes
Lima et al. 2008 [52]	RCT	8–12-year-old patients with new referral for asthma and on no asthma treatment presenting with uncontrolled symptoms	Duration: 7 weeks	Significant improvements in IMRT versus control seen in MIP (∆MIP = 62 cmH_2_0 [IMRT group] vs. 0 cmH_2_0 [control group]; *p* < 0.0001), MEP (∆MEP = 31 cmH_2_0 [IMRT group] vs. 0 cmH_2_0 [control group]), and PEF; daytime and nocturnal symptoms, activity limitation, and asthma attacks
		*N* = 50 (34 females, 16 males)	Frequency: 2 x/week Intensity: 40% MIP	IMRT effects on MIP, MEP, and PEF were generally durable after 7 weeks off IMRT
			Volume: 50 min sessions	No differences between groups in emergency room treatment and hospitalization
			Type: inspiratory pressure threshold resistance	
			Equipment: respironics threshold IMT	
			(Control intervention: education without IMRT; all participants started monthly visits and asthma education; participants could be started on ICS or ICS/LABA and adjusted monthly)	

Elnaggar 2020 [53]	RCT	12–16-year-old patients with past diagnosis of asthma, clinically stable asthma on daily controller	Duration: 12 weeks	Significant improvement in IMRT vs. placebo seen in FEV_1_ (*p* = 0.003), FVC (*p* = 0.001), FEV_1_/FVC (*p* = 0.004), MIP (∆MIP = 17 cmH_2_0 [IMRT group] vs. 11 cmH_2_0 [placebo group]; *p* = 0.002), MEP (∆MEP = 19 cmH_2_0 [IMRT group] vs. 11 cmH_2_0 [placebo group]; *p* = 0.004), and asthma control test score (*p* = 0.001) adjusted to the pretreatment values
		*N* = 31 (11 females, 20 males)	Frequency: 3 x/week	
			Intensity: 40% MIP, reassessed every week	
			Volume: 15 breaths with 10 s rest intervals for 15 min + continuous breathing for 5 min for endurance (total: 20 min sessions)	
			Type: inspiratory pressure threshold resistance	
			Equipment: respironics threshold IMT	
			(Placebo control: IMT at 5% MIP and similar frequency, volume, and duration)	
			Both groups received a conventional respiratory rehabilitation program	

*Note*. RCT–randomized controlled trial, IMT–inspiratory muscle training, ICS–inhaled corticosteroid, LABA–long-acting beta agonist, MIP–maximum inspiratory pressure, MEP–maximum expiratory pressure, PEF–peak expiratory flow, and SABA–short-acting beta agonist.

**Table 6:** Inspiratory muscle rehabilitation training (IMRT) in children with obesity.

Citation	Study Design	Population	Intervention	Outcomes
LoMauro et al. 2016 [61]	Before-and-after trial	12–17-year-old children with obesity (BMI *z* > 2) enrolled in a multidisciplinary body weight reduction program	Duration: 3 weeks	Body weight reduction program with IMRT was associated with improved exercise performance (peak work rate increased by 26 W; *p* = 0.003) and lung and chest wall volume recruitment (inspiratory capacity reduced by 290 ± 550 mL)
		(Mean BMI = 36 kg/cm^2^)	Frequency: 5 x/week	Dyspnea and leg discomfort during exercise were reduced
		*N* = 11 males	Intensity: 50–60% MVV	FVC improved by 4% predicted (*p* = 0.019)
			Volume: 12–18 min sessions, 25 inspirations, 1 session/d	During exercise, abdominal ribcage hyperinflation was delayed and led to
			Type: eucapnic hyperventilation	15% increased exercise capacity and reduced dyspnea at high workloads (*p* < 0.05) without ventilatory and metabolic changes
			Equipment: Spiro 141 Tiger®	MIP and MEP not reported
			All participants underwent a body weight reduction program including	
			energy-restricted diet, psychological and nutritional counseling and aerobic physical activity	

Salvadego et al. 2017 [59]	Non-randomized parallel group trial	15–19-year-old sedentary adolescents with a BMI >97^th^ percentile enrolled in a multidisciplinary body weight reduction program	Duration: 3 weeks	IMRT group had significantly greater PEF (∆PEF = 13% predicted [IMRT group] vs. 3% predicted [control group]), reduced oxygen cost of exercise and dyspnea ratings during exercise completed above the gas exchange threshold compared with the control group
		(mean BMI ≈ 39 kg/cm^2^)	Frequency: 5 x/week	MIP and MEP not reported
		*N* = 17 males	Intensity: 50–60% MVV	
			Volume: 12–18 min sessions, 25 inspirations, 1 session/d	
			Type: eucapnic hyperventilation	
			Equipment: Spiro Tiger®	
			(Control: No IMRT)	
			All participants underwent a body weight reduction program including	
			energy-restricted diet, psychological and nutritional counseling and aerobic physical activity	

Alemayehu et al. 2018 [60]	RCT	15–18-year-old sedentary adolescents with a BMI >97^th^ percentile enrolled in a multidisciplinary body weight reduction program	Duration: 3 weeks	IMRT group had significantly greater improvement in time to exhaustion and peak exercise intensity during a treadmill test
		(Mean BMI = 40 kg/cm^2^)	Frequency: 5 x/week	IMRT had greater reduction in heart rate and oxygen cost of exercise below and above the gas exchange threshold and in dyspnea ratings above the gas exchange threshold
		*N* = 16 males	Intensity: 50–60% MVV	MIP and MEP not reported
			Volume: 12–18 min sessions, 25 inspirations, 1 session/d	
			Type: eucapnic hyperventilation	
			Equipment: Spiro Tiger®	
			(Control: no IMRT)	
			All participants underwent a body weight reduction program including	
			energy-restricted diet, psychological and nutritional counseling and aerobic physical activity	

*Note*. BMI–body mass index, MVV–maximal voluntary ventilation, FVC–forced vital capacity, and PEF–peak expiratory flow.

## Data Availability

Data sharing is not applicable to this article as no new datasets were created or analyzed in this study.

## Supplementary Materials

Supplementary Materials Scoring and quality of studies as well as adverse events for randomized controlled trials, pre-post intervention trials, and case reports/series are reported in Supplemental Tables 1, 2, and 3, respectively.
