# Supplementary material for: Inspiratory Muscle Rehabilitation Training in Pediatrics: What Is the Evidence?
Source: Can Respir J. 2022 Aug 18;2022:5680311. doi: 10.1155/2022/5680311 (PMC9410970; doi:10.1155/2022/5680311)
Supplement: Supplementary Materials — Scoring and quality of studies as well as adverse events for randomized controlled trials, pre-post intervention trials, and case reports/series are reported in Supplemental Tables 1, 2, and 3, respectively. [file 5680311.f1.docx]

**Supplemental Table 1:** Scoring and quality of randomized and non-randomized controlled trials. Adverse events (if reported) are included for each trial.

|  | Wells 2005 | Kilding 2010 | Lemaitre 2013 | Lomax 2019 | Okrzymowska 2019 | Vasickova 2017 | Mackala 2019 | Martin 1986 | Topin 2002 | Yeldan 2008 | Lima 2008 | Elnaggar 2020 | Salvadego 2017 | Alemayehu 2018 |
| --- | --- | --- | --- | --- | --- | --- | --- | --- | --- | --- | --- | --- | --- | --- |
| 1. Was the study described as randomized, a randomized trial, a randomized clinical trial, or an RCT? | Yes | Yes | No | Yes | Yes | Yes | Yes | Yes | Yes | No | Yes | Yes | No | Yes |
| 2. Was the method of randomization adequate (i.e., use of randomly generated assignment)? | Yes | Yes | No | Yes | Yes | Yes | Yes | Yes | Yes | No | Yes | Yes | NA | Yes |
| 3. Was the treatment allocation concealed (so that assignments could not be predicted)? | No | Yes | No | No | No | Yes | No | No | Yes | Yes | No | Yes | No | No |
| 4. Were study participants and providers blinded to treatment group assignment? | No | Yes | No | No | No | Yes | No | No | Yes | Yes | No | Yes | No | No |
| 5. Were the people assessing the outcomes blinded to the participants' group assignments? | CD | CD | CD | CD | CD | Yes | CD | CD | Yes | Yes | No | Yes | No | No |
| 6. Were the groups similar at baseline on important characteristics that could affect outcomes (e.g., demographics, risk factors, co-morbid conditions)? | Yes | Yes | Yes | Yes | Yes | Yes | Yes | Yes | Yes | Yes | Yes | Yes | Yes | Yes |
| 7. Was the overall drop-out rate from the study at endpoint 20% or lower of the number allocated to treatment? | Yes | Yes | Yes | Yes | No | No | No | CD | Yes | Yes | Yes | Yes | Yes | Yes |
| 8. Was the differential drop-out rate (between treatment groups) at endpoint 15 percentage points or lower? | Yes | Yes | Yes | CD | CD | Yes | No | CD | Yes | Yes | Yes | Yes | Yes | Yes |
| 9. Was there high adherence to the intervention protocols for each treatment group? | Yes | Yes | Yes | CD | CD | Yes | CD | CD | Yes | CD | Yes | Yes | Yes | Yes |
| 10. Were other interventions avoided or similar in the groups (e.g., similar background treatments)? | Yes | Yes | Yes | Yes | Yes | Yes | Yes | Yes | Yes | No | Yes | Yes | Yes | Yes |
| 11. Were outcomes assessed using valid and reliable measures, implemented consistently across all study participants? | Yes | Yes | Yes | Yes | Yes | Yes | Yes | Yes | Yes | Yes | Yes | Yes | Yes | Yes |
| 12. Did the authors report that the sample size was sufficiently large to be able to detect a difference in the main outcome between groups with at least 80% power? | No | No | No | No | No | No | No | No | No | No | Yes | Yes | Yes | Yes |
| 13. Were outcomes reported or subgroups analyzed prespecified (i.e., identified before analyses were conducted)? | Yes | Yes | Yes | Yes | Yes | Yes | Yes | Yes | Yes | Yes | Yes | Yes | Yes | Yes |
| 14. Were all randomized participants analyzed in the group to which they were originally assigned, i.e., did they use an intention-to-treat analysis? | Yes | Yes | Yes | Yes | Yes | Yes | Yes | Yes | Yes | Yes | Yes | Yes | Yes | Yes |
| *Overall rating* | Good | Good | Fair | Fair | Fair | Good | Fair | Fair | Good | Good | Good | Good | Fair | Good |
| *Adverse events* | CD | CD | CD | CD | 2 dropped out from IMT grp because of declining health | CD | CD | CD | CD | No side effects reported | CD | No adverse events reported | No side effects reported | No side effects reported |

CD: cannot determine

**Supplemental Table 2:** Scoring and quality of pre-post design intervention trials. Adverse events (if reported) are included for each trial.

|  | Dimarco 1985 | Takaso 2010 | Smith 2017 | LoMauro 2016 |
| --- | --- | --- | --- | --- |
| 1. Was the study question or objective clearly stated? | Yes | Yes | Yes | Yes |
| 2. Were eligibility/selection criteria for the study population prespecified and clearly described? | Yes | Yes | Yes | Yes |
| 3. Were the participants in the study representative of those who would be eligible for the test/service/intervention in the general or clinical population of interest? | Yes | Yes | Yes | Yes |
| 4. Were all eligible participants that met the prespecified entry criteria enrolled? | Yes | Yes | Yes | Yes |
| 5. Was the sample size sufficiently large to provide confidence in the findings? | CD | CD | CD | CD |
| 6. Was the test/service/intervention clearly described and delivered consistently across the study population? | Yes | Yes | Yes | Yes |
| 7. Were the outcome measures prespecified, clearly defined, valid, reliable, and assessed consistently across all study participants? | Yes | Yes | Yes | Yes |
| 8. Were the people assessing the outcomes blinded to the participants' exposures/interventions? | No | No | No | No |
| 9. Was the loss to follow-up after baseline 20% or less? Were those lost to follow-up accounted for in the analysis? | Yes | Yes | Yes | Yes |
| 10. Did the statistical methods examine changes in outcome measures from before to after the intervention? Were statistical tests done that provided p values for the pre-to-post changes? | Yes | Yes | Yes | Yes |
| 11. Were outcome measures of interest taken multiple times before the intervention and multiple times after the intervention (i.e., did they use an interrupted time-series design)? | No | No | No | No |
| 12. If the intervention was conducted at a group level (e.g., a whole hospital, a community, etc.) did the statistical analysis take into account the use of individual-level data to determine effects at the group level? | NA | NA | NA | NA |
| *Overall rating* | Fair | Fair | Fair | Fair |
| *Adverse events* | No adverse events | CD | CD | CD |

CD: cannot determine, NA: not applicable

**Supplemental Table 3:** Scoring and quality of case reports/case series. Adverse events (if reported) are included for each trial.

|  | Jones 2014 | Crisp 2020 |
| --- | --- | --- |
| 1. Was the study question or objective clearly stated? | Yes | Yes |
| 2. Was the study population clearly and fully described, including a case definition? | Yes | Yes |
| 3. Were the cases consecutive? | CD | NA |
| 4. Were the subjects comparable? | Yes | NA |
| 5. Was the intervention clearly described? | Yes | Yes |
| 6. Were the outcome measures clearly defined, valid, reliable, and implemented consistently across all study participants? | Yes | Yes |
| 7. Was the length of follow-up adequate? | Yes | Yes |
| 8. Were the statistical methods well-described? | Yes | Yes |
| 9. Were the results well-described? | Yes | Yes |
| *Overall rating* | Fair | Fair |
| *Adverse events* | No adverse events or side effects | No adverse events or side effects |

CD: cannot determine, NA: not applicable
